# Helical Tomotherapy Combined with Capecitabine in the Preoperative Treatment of Locally Advanced Rectal Cancer

**DOI:** 10.1155/2014/352083

**Published:** 2014-05-06

**Authors:** Ming-Yii Huang, Chin-Fan Chen, Chun-Ming Huang, Hsiang-Lin Tsai, Yung-Sung Yeh, Cheng-Jen Ma, Chan-Han Wu, Chien-Yu Lu, Chee-Yin Chai, Chih-Jen Huang, Jaw-Yuan Wang

**Affiliations:** ^1^Department of Radiation Oncology, Kaohsiung Medical University Hospital, Kaohsiung 807, Taiwan; ^2^Department of Radiation Oncology, Faculty of Medicine, College of Medicine, Kaohsiung Medical University, Kaohsiung 807, Taiwan; ^3^Cancer Center, Kaohsiung Medical University Hospital, Kaohsiung 807, Taiwan; ^4^Division of Gastrointestinal and General Surgery, Department of Surgery, Kaohsiung Medical University Hospital, Kaohsiung 807, Taiwan; ^5^Graduate Institute of Clinical Medicine, College of Medicine, Kaohsiung Medical University, Kaohsiung 807, Taiwan; ^6^Division of General Surgery Medicine, Department of Surgery, Kaohsiung Medical University Hospital, Kaohsiung 807, Taiwan; ^7^ School of Medical and Health Sciences, Fooyin University, Kaohsiung 831, Taiwan; ^8^Department of Medical Research, Fooyin University Hospital, Pingtung 928, Taiwan; ^9^Department of Internal Medicine, Faculty of Medicine, College of Medicine, Kaohsiung Medical University, Kaohsiung 807, Taiwan; ^10^Division of Gastroenterology, Department of Internal Medicine, Kaohsiung Medical University Hospital, Kaohsiung 807, Taiwan; ^11^Department of Pathology, Kaohsiung Medical University Hospital, Kaohsiung 807, Taiwan; ^12^Department of Pathology, Faculty of Medicine, College of Medicine, Kaohsiung Medical University, Kaohsiung 807, Taiwan; ^13^Department of Surgery, Faculty of Medicine, College of Medicine, Kaohsiung Medical University, Kaohsiung 807, Taiwan; ^14^Graduate Institute of Medicine, College of Medicine, Kaohsiung Medical University, Kaohsiung 807, Taiwan; ^15^Department of Medical Genetics, College of Medicine, Kaohsiung Medical University, Kaohsiung 807, Taiwan

## Abstract

The aim of this study was to evaluate the efficacy of helical tomotherapy plus capecitabine as a preoperative chemoradiotherapy (CRT) in patients with locally advanced rectal cancer (LARC). Thirty-six LARC patients receiving preoperative CRT were analyzed. Radiotherapy (RT) consisted of 45 Gy to the regional lymph nodes and simultaneous-integrated boost (SIB) 50.4 Gy to the tumor, 5 days/week for 5 weeks. Chemotherapy consisted of capecitabine 850 mg/m^2^, twice daily, during the RT days. Patients underwent surgery 6–8 weeks after completion of CRT. Information was collected for patient characteristics, treatment response, and acute and late toxicities. Grade 3/4 (G3+) toxicities occurred in 11.1% of patients (4/36). Sphincter preservation rate was 85.2% (23/27). Five patients (14.3%) achieved pathological complete response. Tumor, nodal, and ypT0-2N0 downstaging were noted in 60% (21/35), 69.6% (16/23), and 57.1% (20/35). Tumor regression grade 2~4 was achieved in 28 patients (80%). After a median follow-up time of 35 months, the most common G3+ late morbidity was ileus and fistula (5.7%, 2/35). The study showed that capecitabine plus helical tomotherapy with an SIB is feasible in treatment of LARC. The treatment modality can achieve a very encouraging sphincter preservation rate and a favorable ypT0-2N0 downstaging rate without excessive toxicity.

## 1. Introduction

Since randomized studies have demonstrated that the use of preoperative chemoradiotherapy (CRT) can achieve less acute toxicity and better sphincter preservation rates and reduce the risk for local recurrence, neoadjuvant treatment has become a standard treatment modality in locally advanced rectal cancer (LARC) [1, 2]. In comparison to preoperative radiotherapy (RT) alone, the results of two randomized trials, the European Organization for Research and Treatment of Cancer 22921 trial and the Fédération Francophone de Cancérologie Digestive 92933 trial, have demonstrated that adding fluorouracil-based chemotherapy to RT preoperatively can achieve better local control and downstaging in LARC [3, 4]. As a result, preoperative 5-fluorouracil- (5-FU-) based chemotherapy, in combination with RT, has become the accepted CRT regimen in the neoadjuvant treatment of LARC [2].

Despite the advantages of CRT described above, this therapy has been associated with significant toxicities. For example, in a study by the German Rectal Cancer Study Group, acute and late grade 3/4 (G3+) toxicities were noted in 27% and 14% of LARC patients following 5-FU chemotherapy [1]. Previous studies have shown that replacement of infusional 5-FU with oral capecitabine decreased these toxicities and the possible complications accompanying the use of a venous access device [5, 6]. However, the possible morbidity related to RT remains another major concern in LARC patients.

For all organs at risk (OAR) in RT, the small bowel (SB) is a radiosensitive organ. Previous studies have demonstrated the irradiated volume of SB as a predictive key factor for gastrointestinal (GI) toxicities [7, 8]. The TomoTherapy Hi-Art II System (TomoTherapy Inc., Madison, WI), which fully integrates intensity-modulated radiation therapy (IMRT) and image-guided radiation therapy (IGRT) by combining a linear accelerator, a megavolt (MV) computed tomography (CT) scanner, and a multileaf collimator system, was developed [9]. This combination allows for a more precise delineation of the planning target volume (PTV) and the most accurate irradiation delivery possible. Helical tomotherapy has been clinically shown to decrease the irradiated volume of normal tissue during high-dose RT compared with conventional RT for LARC [10].

Few studies to date have focused on the clinical application of preoperative helical tomotherapy with capecitabine in LARC. The aim of this observational study was to evaluate the efficacy and safety of helical tomotherapy combined with capecitabine as a neoadjuvant treatment in patients with LARC.

## 2. Materials and Methods

### 2.1. Patients

Between January 2008 and December 2012, 36 patients with LARC (T3/T4 disease or any clinically positive N-stage) located within 10 cm from the anal verge were enrolled in the study. This study was approved by the Ethics Committee of Kaohsiung Medical University Hospital in 2007. The details of the baseline assessment before CRT have been previously reported [6]. Informed consent was obtained from all patients.

### 2.2. Concurrent Chemoradiotherapy

All patients were treated with capecitabine (850 mg/m^2^, twice daily, 5 days/week, during the days when RT was administered). RT was carried out using the TomoTherapy Hi-Art II System. All patients underwent planning computed tomography (CT) using a helical CT scanner (Philips, Brilliance 16CT) with a 3 mm slice thickness. Patients were asked to urinate and then drink 250 mL of water 30 minutes before the planned CT and each treatment session.

The GTV was contoured on the Philips Pinnacle treatment planning system (version 8.0; ADAC Laboratories, Milpitas, CA), taking into consideration all clinical information, including digital rectal examination, endoscopy, and all imaging to identify the primary tumor and enlarged regional lymph nodes, with generous coverage to the adjacent presacral space. The GTV-tumor (GTV-T) and GTV-node (GTV-N) were delineated using information from the diagnostic CT and magnetic resonance imaging (MRI). The clinical target volume (CTV) included the GTV-T and GTV-N (if any), the presacral nodes, the complete mesorectum, and the common and internal iliac lymph nodes. The MRI (axial T2 weighted turbo spin echo; 3 mm slice thickness) was used as reference radiologic image to delineate the primary tumor and its mesentery. Lymph nodes along the internal iliac and inferior mesenteric vessels were delineated on CT scan (window, 500; level, 750).

The CTV_45 Gy included a minimum of 2 cm of normal rectum beyond the GTV (primary tumor) in addition to the entire mesorectum and the internal iliac, presacral, and lower common iliac lymph nodes up to the sacral promontory and inferiorly at least to the anal-rectal junction. RT was delivered in 25 fractions, 5 days/week, for 5 weeks (1.8 Gy/fraction). All patients received a simultaneously administered simultaneous-integrated boost (SIB) to 50.4 Gy applied to their primary tumor and gross nodal disease (CTV_50.4 Gy). The CTV_45 Gy was expanded 1 cm toward the plan target volume (PTV_45 Gy), and a less conservative CTV-PTV margin of 0.5 cm was applied for the CTV_50.4 Gy. PTV was subtracted away from the skin 3 mm for this treatment. Before each treatment session, patients underwent scanning using the integrated MV-CT modality and were repositioned after coregistration of these images with the planning kV-CT scan. The small bowel, bladder, and femoral head were delineated as OAR. The entire bladder and individual loops of small bowel and their mesentery were contoured from mid L4 to the lowest extent in the pelvis, and the bladder was fully contoured. The goals were to give at least 95% of the prescribed dose to at least 95% of the PTV, while minimizing the volume of small bowel receiving 15 Gy. Limited volumes of small bowel volume were allowed to exceed 45 Gy if adjacent to the boost volume, but not to exceed 50 Gy. The irradiated volume of bladder that received more than 21 Gy was kept to less than 50% or lower if possible. The maximum bladder dose was kept from exceeding 50 Gy. The proximal femora were constrained from receiving more than 40 Gy.

Patients were evaluated weekly during the course of CRT to assess the acute toxicity. Acute toxicity was monitored using the National Cancer Institute Common Toxicity Criteria, version 3.0 (http://ctep.cancer.gov/reporting/ctc.html; accessed in December 2011). Principles for dose modification or discontinuation of CRT have been previously reported [6].

### 2.3. Surgery

Patients underwent surgery 6–8 weeks after completion of CRT. All operations were carried out by the two specialist colorectal surgeons at a single institution. Anal sphincter-sparing surgery was performed whenever possible with primary anastomosis and/or temporary diverting stomas [11]. Perioperative complications (within 60 days after surgery) [12] were confirmed either clinically or radiographically using CT scan. Patients were followed monthly in the postoperative first year, every 3 months for 3 years, and twice a year thereafter. Abdominal CT scan was performed when warranted by clinical symptoms or examinations.

### 2.4. Late Morbidity

Late toxicity was recorded according to the objective criteria of the Radiation Therapy Oncology Group (RTOG)/European Organization for Research and Treatment of Cancer (EORTC) scale with respect to the late adverse effects of RT [13]. Late toxicity has been scored 61 days after the surgery [12]. Late severe adverse effect (SAE) was recognized when it met any of the following criteria: toxic death, G3+ toxicities, and requiring major surgical intervention or hospitalization.

### 2.5. Study Endpoints

All patient data were collected using hospital electronic record and chart review. The primary endpoint was to determine the efficacy of the treatment modality in LARC. Efficacy was assessed by determining the results of pathological complete response (pCR), tumor (T) downstaging rate, nodal (N) downstaging, ypT0-2N0 downstaging, tumor regression grade (TRG), and sphincter preservation rate for low-lying rectal cancer. A pCR was defined as the absence of any viable tumor cell in the tumor specimen, including regional lymph nodes. T and N downstaging were defined as reductions in T and N stages by at least one level. The TRG of the primary tumor was determined by the same pathologist based on the tumor regression grading system initially described by Dworak et al. [14]. The secondary endpoint was to determine the safety of the treatment modality in LARC. Safety was assessed mainly by the proportion of patients who experienced G3+ acute toxicities during CRT, perioperative complications, and late SAE.

### 2.6. Statistical Analysis

All data were analyzed using the Statistical Package for the Social Sciences, version 18.0 (SPSS Inc., Chicago, IL). An independent *t*-test was used for comparison of continuous variables. Categorical data were analyzed by the Pearson chi-square test or Fisher's exact test (two-sided). A *P* value less than 0.05 was considered statistically significant.

## 3. Results

### 3.1. Patients Characteristics

Thirty-six (19 men and 17 women; median age, 63 years (range, 34–81 years)) patients were analyzed, and their characteristics are summarized in Table 1. A full RT dose and capecitabine dose were delivered in 94.4% (34/36) and 88.9% (32/36) of patients, respectively.

### 3.2. Acute Toxicities

All patients in the study were assessable for acute toxicities, which are listed in Table 2. The most common acute adverse events encountered were dermatitis (75%), followed by diarrhea (69.5%). Of all the patients with dermatitis, 77.8% had low-lying rectal tumors. Four patients (11.1%) developed G3 acute toxicities, and all of the G3 acute toxicities in the study were diarrhea. There was no G3+ hematologic toxicity and no G4 nonhematologic toxicity reported. Fortunately, all of the severe toxicities encountered in the study could be ameliorated after adequate conservative treatment. No patient in this study withdrew from CRT because of any intolerable toxicity. All patients finished their preoperative CRT without treatment interruptions.

### 3.3. Sphincter Preservation

After completion of CRT, 35 patients underwent definitive surgery. One patient declined surgery after CRT and was excluded from the assessment of efficacy, perioperative complications, and late morbidity. Surgery was performed after a median interval of 42 days (range: 27–56 days). The types and numbers of operations performed are listed in Table 1. The surgical result was classified as R0 resection in 33 patients (94.3%). Twenty-seven of the 36 patients (75%) had low-lying tumors (tumor located ≤5 cm from the anal verge (AV)). Among the 27 patients with low-lying tumors, 23 (85.2%) were able to undergo the sphincter-sparing procedure. Seventeen of the 23 patients (73.9%) receiving sphincter-preserving surgery also underwent diverting stomas during the same operation.

### 3.4. Pathological Response

The objective pathologic response and the results of TRG are listed in Table 3. pCR was achieved in 5 patients (14.3%). T downstaging rate was 60%, and N downstaging in clinical N1-2 patients was achieved in 69.6% of the patients. Eleven of the 12 patients (91.7%) with clinical N0 showed no node metastasis after CRT. Furthermore, 20 patients (57.1%) achieved the ypT0-2N0 downstaging. TRG 2~4 was noted in 80% of the patients (28/35). Twenty of the 28 patients (71.4%) categorized as “major responders” (TRG, 2~4) in the study achieved T downstaging, compared with one of the remaining 7 patients (14.3%) in “minor responders” (TRG, 0~1) showing T downstaging (*P* = 0.010).

### 3.5. Perioperative Morbidity and Mortality

Among the 29 patients who underwent LAR, 2 patients (6.9%) experienced anastomotic leakage. Subsequently, both patients underwent a diverting colostomy for fecal diversion. Pelvic abscess manifested as intermittent fever was found in one patient with underlying diabetes mellitus 42 days postoperatively. This 69-year-old male received percutaneous CT-guided drainage of the pelvic abscess. Perineal wound complications were noted in 2 patients who received abdominoperineal resection (APR). All of them made an uneventful recovery after the treatments. Neither life-threatening complications nor any treatment-related deaths occurred within the 60 days following surgery.

### 3.6. Late Morbidity

The median follow-up time was 35 months (range 15–65 months). The incidence of late SAE was 14.3% (5/35), and gastrointestinal (GI) adverse effects were the most common late SAE in the study (Table 2). The incidence of grade 3 SB obstruction was 5.7% (2/35, at 7th and 14th month postoperatively) in the study. One of the 2 patients required surgical intervention due to failed conservative treatment. Both had an uneventful recovery after the treatment. Four of the 35 patients (11.4%) encountered grade 2 anastomotic stenosis and required anal bougination during the follow-up period. One 66-year-old patient experienced symptomatic colonic obstruction secondary to grade 3 sigmoid colon stenosis at the 17th month postoperatively. Her condition improved soon after the endoscopic balloon dilatation procedure. Two patients (5.7%) suffered from grade 2 colitis during the follow-up time. Fortunately, their conditions were reversible after treatment. Besides, two patients (5.7%) encountered grade 2 chronic diarrhea and their symptoms were manageable by antidiarrheal agents. Grade 2 fecal incontinence was seen in 1 patient (2.9%) who received radical proctectomy with coloanal anastomosis. Colovaginal fistula was found in two patients (5.7%) at 4th and 11th month postoperatively. Subsequently both patients underwent fistulectomy and had a smooth postoperative course. Four patients experienced grade 2 cystitis and required further treatment. Grade 2 distal ureteral stenosis with hydronephrosis was noted in 3 patients (8.6%). However, all 3 patients improved soon after double-J catheter implantation and did not require any further operation.

### 3.7. Follow-Up and Outcome

Nine out of 35 postoperative patients did not receive adjuvant chemotherapy. Four patients with pCR and four patients with pathological stage 1 declined adjuvant chemotherapy. The remaining one (pT4aN0M0) postponed the chemotherapy due to perineal wound complications following APR. Since lung metastases developed 3 months after surgery, she received systemic chemotherapy for progression of disease. All the 9 patients with pathologically confirmed positive lymph node disease received adjuvant chemotherapy. Of the 36 patients enrolled in the study, local recurrence or systemic progression of disease was observed in 16 patients (44.4%). Six patients developed local recurrence of the disease. There were five in-field recurrences and one out of field recurrence. Seven patients developed liver metastases; 2 patients developed lung metastases and the remaining one was found to have bone as the first site for cancer metastasis. To date, six patients have died of metastatic disease after 55, 53, 44, 42, 23, and 16 months, respectively.

## 4. Discussion

To the best of our knowledge, this is the first comprehensive study regarding the efficacy and safety of preoperative helical tomotherapy plus capecitabine in LARC.  Table 4 summarizes the results from previously published studies regarding the efficacy of preoperative RT plus capecitabine in the treatment of LARC [5, 12, 15–26]. It shows pCR rates ranging from 6.7 to 31% in other studies compared with 14.3% in the study. T downstaging rate was 41.9–76.7% in the previous studies compared with 60% in the study. Besides, N downstaging rate was 50–87.5% in the previous studies compared with 69.6% in the study. For patients with low-lying rectal cancer, the sphincter preservation rate in the study was 85.2%, which seems higher than those in other studies (14.3–67.7%). Finally, the ypT0-2N0 downstaging rate was 57.1% in the study compared with 41.2–50% in the previous studies.  Table 5 shows the comparison of acute toxicities and perioperative complications [5, 12, 15–26]. G3+ acute toxicities were noted in 5–15% of patients in other studies compared with 11.1% in the study. Besides, the incidence of acute G3+ diarrhea in the study was 11.1%, with a comparable result to most other published studies (2–35.5%). In consideration of the relation between RT and dermatitis, it is worth noting that no patient in the present study encountered G3 dermatitis compared with 0–9% in other studies. In addition, 10.3% of the patients in the study experienced anastomotic leakage and/or pelvic abscess after LAR, compared with 2.4–30.8% in the previous studies. The incidence of wound complication was 5.7% in the study, compared to 0.9–37.9% in other studies.

Previous studies have reported that the incidence of G3+ acute toxicities was 5–15% [5, 12, 15–26], which is consistent with our result (11.1%). However, absence of G3 dermatitis in the study, compared with other studies, was an encouraging result. It has been noted that dermatitis is a complication highly related to irradiation. Most patients enrolled in the study had low-lying rectal tumors (75%); however, no patients experienced G3 dermatitis. Our results compare favorably with the incidence of G3 dermatitis reported by Kim et al. (3.3%, 3-field technique, 42% patients with low-lying rectal tumors) [5]; De Paoli et al. (4%, 3- or 4-field technique, 68% patients with low-lying rectal tumors) [18]; Park et al. (3%, 3- or 4-field technique, 60% patients with low-lying rectal tumors) [12]; and Li et al. (3.2%, IMRT, 85.7% patients with low-lying rectal tumors) [26]. Although exact reasons for the difference are not clear, we propose that absence of G3 dermatitis in the study might be due to an optimized dose distribution and a decreased radiation dose to skin by applying helical tomotherapy. Further prospective randomized trials may better define the role of helical tomotherapy in this clinical setting.

Regarding the incidence of anastomotic leakage or pelvic abscess after LAR, the study showed a comparable result (10.3%) with that reported by the German Rectal Cancer Study Group (11%) [1]. We noticed that both patients experiencing anastomotic leakage had no protective stoma in initial LAR. It is worth noting that none of the 20 patients with a temporary diverting stoma in comparison with two of the remaining 9 patients (17.6%) without a diverting stoma experienced anastomotic leakage (*P* = 0.085). Although no significant difference was noted in the study, the patients who underwent sphincter-sparing surgery with a stoma seemed less likely to experience the complication. One meta-analysis study has pointed out that a diverting stoma can reduce the rate of clinically relevant anastomotic leakages and is thus recommended in surgery for low rectal cancers [27]. However, the role of a diverting stoma construction in patients undergoing preoperative CRT and a subsequent LAR for rectal cancer is still controversial. In a prospective trial for anastomotic complication survey after preoperative CRT and subsequent LAR without a diverting stoma, Huh et al. reported a relatively low anastomotic complication rate in patients with low-lying rectal cancer [28]. Thus they suggested that a diverting stoma is not necessary when performing LAR and handsewn coloanal anastomosis for lower rectal cancer. In another retrospective case series about neoadjuvant CRT for rectal cancer, Tsikitis et al. reported that there were 4.1% anastomotic leakage rate in patients receiving LAR and a diverting stoma and suggested that a diverting stoma could mitigate the serious sequels of anastomotic leakage after LAR in patients who are preoperatively irradiated for low-lying rectal cancer [29]. We believe that more evidence from further studies is still required to determine whether a routine diverting stoma is necessary in surgical management of low-lying rectal cancer after CRT.

Several indices of efficacy, such as T/N downstaging, pCR, and ypT0-2N0, have been demonstrated as prognostic factors in LARC patients [30–32]. It is noteworthy that our ypT0-2N0 downstaging rate compares favorably with other studies using capecitabine plus RT (ypT0-2N0 41.2–50%) [5, 12, 15, 20, 23–25]. Furthermore, other indices of tumor response (T/N downstaging, pCR) are comparable to other studies (Table 4) [5, 12, 15–26]. Indeed, optimization of either RT or chemotherapy is known to be a practicable method for improving tumor response or minimizing treatment-related toxicity. In a study of applying image-guided tomotherapy in preoperative CRT for rectal cancer, Passoni et al. have confirmed the feasibility of adding a boost to the gross tumor volume (total tumor dose 45.6 Gy) while remaining concomitant to the oxaliplatin-based chemotherapy [33]. A promising tumor response with an acceptable toxicity was showed in the trial (pCR 30.4%, T downstaging 69.6%, G3+ acute toxicity 12%). In one phase II trial of preoperative helical tomotherapy for rectal cancer, De Ridder et al. demonstrated that tomotherapy allows delivery of SIB of 55.2 Gy to the primary tumor, without increasing the irradiated volume of SB or acute toxicity [34]. Additionally, it is interesting to note that the incidence of G3 acute toxicity in the present study (11.1%) compares favorably with that reported in our previous study (19.1%), using conventional RT technique plus the same capecitabine regimen [6]. Thus the relatively acceptable toxicity profile in the study may suggest the possibility of a RT dose escalation to the primary tumor by helical tomotherapy, subsequently achieving a more favorable treatment effect.

For tumors located within 5 cm of the AV, where an APR was traditionally considered necessary, the sphincter preservation rate in the study was higher than those reported in other studies (Table 4) [5, 12, 15, 18, 20, 21, 23–26]. Although causes of the difference are not clear, the higher sphincter preservation rate might be explained by several reasons. First, most patients in the study were of cT3 stage. Second, a higher dose of capecitabine eventuated in obvious tumor shrinkage. Third, further evolution of the surgical technique was achieved [11]. Finally, it might be due to tumor shrinkage secondary to CRT with helical tomotherapy, which offers optimized target coverage and a more precise irradiation delivery to the gross tumor [10]. Three out of six recurrences were noted at 1 cm, 2 cm, and 3 cm near the anal verge, respectively. Surgical intent for sphincter preservation may explain these three recurrences near anal verge.

The incidence of late SAE (14.3%) was comparable to the study conducted by Sauer et al. (14%) [1]. It has been noted that the symptoms resulting from radiation-related GI toxicity were diarrhea and obstruction due to stenosis or adhesions, as well as bleeding, necrosis, perforation, fistulation, and fecal incontinence [35]. The Stockholm I and II Trials have demonstrated that preoperative RT significantly increased the incidence of SB obstruction [36]. The Dutch colorectal cancer group reported 11% of the preoperatively irradiated patients encountered SB obstruction in a 5-year follow-up [37]. In a phase 3 trial conducted by the German rectal cancer study group, G3+ chronic diarrhea and SB obstruction were noted in 9% of patients, as well as G3+ anastomotic stricture in 4% of patients in a median 46-month follow-up [1]. Another phase 3 trial reported perioperative and late G3+ ileus in 5.6% of patients and G3+ fistula in 1.9% of patients in a median follow-up time of 52 months [12]. Herein, our results of the late GI SAE were comparable to those of other studies. Additionally, no incidence of urinary SAE was noted in the study, compared to 1.4–2% in other studies with CRT [1, 12, 35]. In view of the above reasons, this study confirmed that helical tomotherapy plus capecitabine is feasible for LARC.

Because many variables may influence the results of CRT in LARC patients in different hospitals, we compare the data in the study (tomotherapy group) with those who received nontomotherapy RT (three-field conventional RT) plus capecitabine in our institution. Between January 2008 and December 2012, 60 patients with LARC (39 males and 21 females; median age, 63 years (range, 36–85 years)) received the same capecitabine regimen and nontomotherapy RT preoperatively. Both groups were well matched for mean age (tomotherapy versus nontomotherapy, 64.8 versus 62.5, *P* = 0.345), gender (male, 52.8% versus 65.0%, *P* = 0.236), clinical T stage (cT3, 97.2% versus 91.7%, *P* = 0.405), clinical node metastasis (cN1-2, 63.9% versus 75.0%, *P* = 0.246), and distance from the AV (≤5 cm from the AV, 75.0% versus 66.7%, *P* = 0.389). Thirty-five of the 36 patients in the tomotherapy group (97.2%) and 57 of the 60 patients in the nontomotherapy group (95.0%) received surgery after CRT (*P* = 1.000). No significant differences were found between the two groups for pCR (tomotherapy versus nontomotherapy, 14.3% versus 8.8%, *P* = 0.497), T downstaging (60% versus 61.4%, *P* = 0.893), N downstaging in patients with cN1-2 (69.6% versus 79.1%, *P* = 0.391), ypT0-2N0 (57.1% versus 43.9%, *P* = 0.216), and sphincter preservation rate for low-lying rectal cancer (85.2% versus 80.0%, *P* = 0.749).

Four patients in the tomotherapy group (11.1%) and 10 patients in the nontomotherapy group (16.7%) developed G3 acute toxicities during CRT (*P* = 0.559). One patient in the nontomotherapy group experienced G3 radiation dermatitis while no serious acute perineal skin toxicity in the tomotherapy group was shown. Although there was no statistically significant difference between groups in the G2+ radiation dermatitis, the patients with low-lying rectal cancer in the nontomotherapy group were more likely to encounter the adverse event than the patients with low-lying rectal cancer in the tomotherapy group (13.9% versus 20.0%, *P* = 0.584). After 35-month median follow-up in the tomotherapy group and 27-month median follow-up in the nontomotherapy group, the rates of G3+ late morbidities did not differ significantly (tomotherapy versus nontomotherapy, 14.3% versus 21.1%, *P* = 0.582). Two patients in the tomotherapy group (5.7%) and 6 patients in the nontomotherapy group (10.5%) suffered from G2+ enteritis/colitis (*P* = 0.706). The incidence of G2+ small bowel obstruction was 11.4% in the tomotherapy group and 21.1% in the nontomotherapy group (*P* = 0.273). Although there was no statistically significant difference due to the limited case number, G2+ late GI toxicity was less likely to occur in the tomotherapy group than in the nontomotherapy group.

Although the study has provided important new information regarding helical tomotherapy plus capecitabine in the preoperative treatment of LARC, it does have some limitations. First, the sample size was small. Second, the limited follow-up period (median, 35 months) allowed for the assessment of response rates but not survival rates, for which a longer follow-up period would be required. Third, the acute and late toxicities collected in the study were based on hospital records. This may lead to underestimation of less severe toxicities because these may be ignored by the physicians during the follow-up time. Finally, further prospective randomized controlled studies are still required to identify the differences in efficacy and safety between the CRT regimens.

In conclusion, the present study showed that capecitabine plus helical tomotherapy with an SIB is practicable in LARC patients. Considering the horseshoe-shape form of the PTV, with the small bowel and bladder lying in the middle, IMRT seems to be the treatment of choice. The concave and sharp dose gradients created by IMRT of course are less forgiving than conventional RT plans in terms of treatment uncertainties and require daily accurate positioning, which can be obtained with the recent evolution in IGRT. Tomotherapy offers an elegant way to implement this concept in daily practice because it fully integrates IGRT by means of MV-CT scanning and IMRT by means of dynamic rotational therapy. Another potential advantage of this technique is the possibility to deliver a simultaneous integrated radiation boost on the gross tumor volume. In current study, the treatment modality can achieve a very encouraging sphincter preservation rate for low-lying rectal cancer and a favorable ypT0-2N0 downstaging rate without excessive toxicity. Further prospective randomized trials are still required to define the definite role of neoadjuvant CRT with helical tomotherapy in LARC.

## Figures and Tables

**Table 1:** Characteristics of the studied patients.

Case number	*N* (%)
Age (years) median 63.0 (range, 34–81)	
Gender	
Male	19 (52.8)
Female	17 (47.2)
ECOG^a^ performance status	
0	35 (97.2)
1	1 (2.8)
Distance from anal verge	
≤5 cm	27 (75.0)
>5 cm	9 (25.0)
Clinical tumor stage (T)	
T3	35 (97.2)
T4 (T4a + T4b)	1 (2.8)
Initial nodal stage (N)	
N0	13 (36.1)
N1	15 (41.7)
N2	8 (22.2)
Tumor differentiation	
Well	3 (8.3)
Moderate	29 (80.6)
Poorly	1 (2.8)
Uncertain type	3 (8.3)
Diabetes mellitus	
Yes	9 (25.0)
No	27 (75.0)
Operation methods	
Low anterior resection	14 (38.9)
Radical proctectomy with coloanal anastomosis	15 (41.7)
Abdominoperineal resection	4 (11.1)
Transanal excision	2 (5.6)
No definite surgery	1 (2.8)

Case number	*N* = 27 (%)

Sphincter-preserving surgery (tumor ≤5 cm from anal verge)	
Yes	23 (85.2)
No	4 (14.8)
Follow-up time (months)	
median	35
range	15–65

^a^Eastern Cooperative Oncology Group.

**Table 2:** Acute toxicities, perioperative complications, and late morbidities in patients with locally advanced rectal cancer.

Acute toxicities	*N* = 36 (%)
Grade 3 or 4 toxicities	4 (11.1)
Nausea/vomiting	
Grade 1	5 (13.9)
Grade 2	1 (2.8)
Diarrhea	
Grade 1	11 (30.6)
Grade 2	10 (27.8)
Grade 3	4 (11.1)
Leukopenia	
Grade 1	2 (5.6)
Grade 2	1 (2.8)
Anemia	
Grade 2	3 (8.3)
Frequency/urgency/cystitis	
Grade 1	6 (16.7)
Grade 2	3 (8.3)
Dermatitis	
Grade 1	22 (61.1)
Grade 2	5 (13.9)
Hand-foot syndrome	
Grade 1	1 (2.8)
Grade 2	1 (2.8)

Perioperative complications	*N* = 29 (%)

Anastomotic leakage (after low anterior resection)	2 (6.9)
Pelvic abscess (after low anterior resection)	1 (3.4)

Late morbidities	*N* = 35 (%)

Grade ≥ 3 toxicities	5 (14.3)
Colitis	
Grade 1	3 (8.6)
Grade 2	2 (5.7)
Small bowel obstruction	
Grade 2	2 (5.7)
Grade 3	2 (5.7)
Anastomotic stenosis	
Grade 1	1 (2.9)
Grade 2	4 (11.4)
Grade 3	1 (2.9)
Fistula	
Grade 4	2 (5.7)
Chronic diarrhea	
Grade 1	4 (11.4)
Grade 2	2 (5.7)
Stool incontinence	
Grade 2	1 (2.9)
Ureter adhesion or stricture	
Grade 1	4 (11.4)
Grade 2	3 (8.6)
Cystitis and/or hematuria	
Grade 1	4 (11.4)
Grade 2	4 (11.4)

**Table 3:** Pathological stage and response after preoperative chemoradiotherapy in patients with locally advanced rectal cancer.

Case number			*N* = 35 (%)
Tumor regression grade (TRG)			
Grade 0			2 (5.7)
Grade 1			5 (14.3)
Grade 2			7 (20)
Grade 3			16 (45.7)
Pathological complete response (TRG grade 4)			5 (14.3)
Pathological stage			
Stage 1			15 (42.9)
Stage 2			6 (17.1)
Stage 3			9 (25.7)
ypT0-T2N0			
Yes			20 (57.1)
No			15 (42.9)
Lymphovascular invasion			
Yes			6 (17.1)
No			29 (82.9)
Perineural invasion			
Yes			8 (22.9)
No			27 (77.1)
Pathological T stage			
Downstaging			21 (60.0)
Stable			14 (40.0)
Progressive			0

Case number			*N* = 23 (%)

Pathological N stage (in clinical N1-2 patients)			
Downstaging			16 (69.6)
Stable			6 (26.1)
Progressive			1 (4.3)

Correlation between TRG and pathological downstage (*N* = 35)			
	Major responder^a^	Minor responder^b^	*P*

T downstaging			
Yes	20	1	0.010
No	8	6	
ypT0-2N0			
Yes	19	1	0.027
No	9	6	
Pathologic node metastasis			
Yes	5	4	0.055
No	23	3	
Lymphovascular invasion			
Yes	4	2	0.576
No	24	5	
Perineural invasion			
Yes	4	4	0.033
No	24	3	
N downstaging *n* = 23 (in clinical N1-2 patients)			
Yes	13	3	0.621
No	5	2	

^a^TRG 2, TRG 3, and TRG 4 are recognized as major responders after chemoradiotherapy; ^b^TRG 0 and TRG 1 are recognized as minor responders after chemoradiotherapy.

**Table 4:** Summary of studies showing the efficacy of capecitabine-based preoperative chemoradiotherapy in patients with locally advanced rectal cancer.

Oral capecitabine	Case number	Dose of capecitabine	Dose of RT	pCR (%)	Sphincter preservation (%) (for low-lying rectal tumor)	Tumor (T) downstaging (%)	Nodal (N) downstaging (%) (in clinical N1-2 patients)	ypT0-2N0 (%)
Krishnan et al. (2006) [15]	51	825 mg/m^2^ twice daily, for the duration of RT	3-field technique; 52.5 Gy in 30 fractions, 5 days/wk ∗ 6 wks	17.6	66.7	51	51.7	44

Kim et al. (2006) [5]	97	825 mg/m^2^ twice daily for 14 days followed by a 7-day rest period	3-field technique; 50.4 Gy in 28 fractions, 5 days/wk ∗ 5.5 wks	22.2	66.7	61.1	87.5	44.4

Yerushalmi et al. (2006) [16]	43	825 mg/m^2^ twice daily, 5 days/wk on RT day	3-field technique; 50.4 Gy in 28 fractions, 5 days/wk ∗ 5.5 wks	30.2	N	76.7	N	N

Das et al. (2006) [17]	89	825 mg/m^2^ twice daily, 5 days/wk (65.2%) and 7 days/wk (34.8%) on RT day	3-field technique; 45 Gy in 25 fractions, 5 days/wk ∗ 5 wks, 0–7.5 Gy boost	21.3	N	51.7	N	N

De Paoli et al. (2006) [18]	53	825 mg/m^2^ twice daily, 7 days/wk on RT day	3- or 4-field technique; 50.4 Gy in 28 fractions, 5 days/wk ∗ 5.5 wks	24	58.8	56.9	78.6	N

Craven et al. (2007) [19]	70	900 mg/m^2^ twice daily, 5 days/wk on RT day	3- or 4-field technique; 45 Gy in 25 fractions, 5 days/wk ∗ 5 wks	9.2	N	41.9	N	N

de Bruin et al. (2008) [20]	60	825 mg/m^2^ twice daily, 5 days/wk on RT day	3-field technique or IMRT; 50 Gy in 25 fractions, 5 days/wk ∗ 5 wks	13.3	50	66.7	63.3	45

Bazarbashi et al. (2008) [21]	30	825 mg/m^2^ twice daily, 7 days/wk on RT day	3- or 4-field technique; 50.4 Gy in 28 fractions, 5 days/wk ∗ 5.5 wks	6.7	14.3	53.8	50	N

Dunst et al. (2008) [22]	96	825 mg/m^2^ twice daily, 7 days/wk for the duration of RT	3D-CRT; 50.4 Gy, daily 1.8 Gy ∗ 5-6 wks, 5.4 Gy boost for T4 lesions	6.9	N	67.8	67.2	N

Elshazly et al. (2009) [23]	26	825 mg/m^2^ twice daily, on RT day	3-field technique; 50 Gy in 25 fractions, 5 days/wk ∗ 5 wks	11.5	53.8	53.8	63.6	50

Chan et al. (2010) [24]	34	825 mg/m^2^ twice daily, 5 days/wk on RT day	4-field technique; 50 Gy in 25 fractions, 5 days/wk ∗ 5 wks	20.6	23.1	58.8	N	41.2

Park et al. (2011) [12]	105	825 mg/m^2^ twice daily, 7 days/wk on RT day	3- or 4-field technique; 50 Gy in 25 fractions, 5 days/wk ∗ 5 wks	17.1	67.7	N	N	41.9

Jin et al. (2011) [25]	59	825 mg/m^2^ twice daily, 5 days/wk on RT day	3 or 4-field technique; 50.4 Gy in 28 fractions, 5 days/wk ∗ 5.5 wks	13.6	62.1	57.6	63.4	33.9

Li et al. (2012) [26]	58	825 mg/m^2^ twice daily, 5 days/wk on RT day	IMRT; 50.6 Gy (GTV) and 41.8 Gy (CTV) in 22 fractions, 5 times per wk over 30 days	31.0	66	56.9	79.2	N

The current study (2014)	35	850 mg/m^2^ twice daily, 5 days/wk on RT day	Helical tomotherapy; 45 Gy to the regional lymph nodes and areas at risk for harboring microscopic disease and SIB 50.4 Gy to the gross tumor in 25 fractions, 5 days/wk ∗ 5 wks	14.3	85.2	60	69.6	57.1

CRT: chemoradiotherapy; LARC: locally advanced rectal cancer; RT: radiotherapy; pCR: pathological complete response; Wk: week; N: not reported; 3D-CRT: 3-dimensional conformal radiotherapy; IMRT: intensity-modulated radiotherapy; GTV: gross target volumes; CTV: clinical target volumes; SIB: simultaneous integrated boost.

**Table 5:** Summary of grade 3 or 4 acute toxicities and perioperative complications in locally advanced rectal cancer patients with capecitabine-based chemoradiotherapy.

Oral capecitabine + RT	Grade 3 or 4 acute toxicities (%)						Perioperative complications (%)	
	Overall Gr 3 or 4 toxicities	Diarrhea	Leukopenia	Anemia	Dermatitis	Hand-foot syndrome	Anastomotic leakage or pelvic abscess (after LAR)	Wound complications
Krishnan et al. (2006) [15]	N	2	2^a^	2	9	0	2.6	3.9
Kim et al. (2006) [5]	N	11.3	0	0	3.1	6.2	5.3	1.1
Yerushalmi et al. (2006) [16]	14	2.3	0	0	4.7	2.3	N	N
Das et al. (2006) [17]	5.6	4.5	1.1	0	0	0	N	N
De Paoli et al. (2006) [18]	11.3	1.9	3.8	3.8	3.8	3.8	N	N
Craven et al. (2007) [19]	N	4.3	N				N	
de Bruin et al. (2008) [20]	5	1.7	0	0	3.3	0	4	N
Bazarbashi et al. (2008) [21]	N	35.5	3.2	3.2	N	0	30.8	37.9
Dunst et al. (2008) [22]	N	7	N		1.1	0	N	
Chan et al. (2010) [24]	8.8	8.8	0	0	0	0	N	
Park et al. (2011) [12]	15	N	2.8	5.6	2.8	2.8	2.4	0.9
Jin et al. (2011) [25]	8.2	3.3	3.3	0	0	1.6	N	6.8
Li et al. (2012) [26]	14.3	9.5	1.6^a^	0	3.2	0	N	
The current study (2014)	11.1	11.1	0	0	0	0	10.3	5.7

LARC: locally advanced rectal cancer; CRT: chemoradiotherapy; RT: radiotherapy; Gr: grade; N: not reported; LAR: low anterior resection.

^a^Neutropenia.
